# Sex Differences in the Recurrence Rate and Risk Factors for Primary Giant Cell Tumors Around the Knee in China

**DOI:** 10.1038/srep28173

**Published:** 2016-06-20

**Authors:** Yongcheng Hu, Liming Zhao, Huilin Zhang, Xiuchun Yu, Zhen Wang, Zhaoming Ye, Sujia Wu, Shibing Guo, Guochuan Zhang, Jinghua Wang, Xianjia Ning

**Affiliations:** 1 Department of Orthopedic Oncology, Tianjin Hospital, 406 Jiefang South Road, Tianjin 300210, China; 2 The Graduate School, Tianjin Medical University, 22 Qixiangtai Road, Heping District, Tianjin, 300071, China; 3 Department of Orthopedics, The General Hospital of Jinan Military Commanding Region, 25 Shifan Road, Jinan, Shandong 250031, China; 4 Department of Orthopedics, Xijing Hospital, Forth Military Medical University, No. 15 West Changle Road, Xincheng District, Xi’an, Shaanxi, 710032, China; 5 Centre for Orthopaedic Research, Department of Orthopaedics, The Second Affiliated Hospital of Zhejiang University, School of Medicine, 88 Jiefang Road, Hangzhou, 310008, China; 6 Department of Orthopaedics, Jin Ling Hospital, 305 Zhong Shan East Road, Nanjing 210002, Jiangsu Province, China; 7 Orthopedics Department, Second Affiliated Hospital of Inner Mongolia Medical University, 1 Yingfang Road, Huimin District, Hohhot, 010050, P.R. China; 8 Department of Orthopedic Oncology, The Third Hospital of Hebei Medical University, 139 Ziqiang Rd, Shijiazhuang 050051, China; 9 Department of Epidemiology, Tianjin Neurological Institute & Department of Neurology, Tianjin Medical University General Hospital, 154 Anshan Road, Heping District, Tianjin, 300052, China

## Abstract

Although giant cell tumor of bone (GCTB) is more common in women in Western countries, it tends to be more common in men in Asian countries. We aimed to determine the sex differences in clinical characteristics, local recurrence rate, and relevant risk factors for local recurrence in primary GCTB around the knee. Between March 2000 and June 2014, patients with primary GCTB around the knee were recruited from 7 institutions in China, and 410 patients were included. The age at diagnosis was younger in women than in men (34.0 vs 37.2 years). The local recurrence rates were 23.4% overall, 25.8% in men, and 20.7% in women. Lower local recurrence rates were observed with en-bloc marginal resection in both men (6.9%) and women (3.1%). With tumors located in the distal femur, the local recurrence rate was higher for men than for women (29.1% vs 14.3%, P = 0.025). Local recurrence was significantly associated with the tumor location and surgical operation in men and only surgical operation in women. These findings suggest that more aggressive operations should be considered in men with GCTB in the proximal fibula.

Giant cell tumor of bone (GCTB) accounts for only 3–8% of primary bone tumors in Western populations, compared with 20% in Asian countries^1^,^2^,^3^,^4^,^5^,^6^,^7^. GCTB is particularly likely to occur around the knee (including the distal femur, proximal tibia, proximal fibula, and patella), accounting for >50% of cases^2^,^3^,^8^,^9^,^10^,^11^.

GCTB is one of the most controversial and widely discussed bone tumors, with a reported postoperative recurrence rate of 10–65%^4^,^5^,^12^,^13^,^14^,^15^,^16^. Furthermore, a previous study conducted in China reported a 12.4% local recurrence rate in patients with primary GCTB located in an extremity^6^. However, all the patients collected in these previous reports were from a single institution and followed over a long time interval. Moreover, although GCTB is more likely to occur in women than in men in Western countries^17^,^18^,^19^, several other studies have reported that GCTB predominately occurs in men, with a male/female sex ratio of 1.27–1.77:1^6^,^9^,^20^,^21^,^22^. Nevertheless, the relevant risk factors associated with recurrence of GCTB do not appear to differ by sex.

We aimed to determine the sex differences in clinical characteristics, local recurrence rate, and relevant risk factors for local recurrence among patients with primary GCTB around the knee in China using a multicenter retrospective study and to establish the appropriate individualized surgical operation to reduce the long-term local recurrence rate.

## Results

### Patient Characteristics and Follow-Up

After excluding 100 patients (19.6%) with recurrent tumors that were treated elsewhere, 410 patients (80.4%) with primary GCTB around the knee were recruited (217 [52.9%] men, 193 [47.1%] women; male:female ratio, 1.12:1).

The median follow-up durations were 55 months (interquartile range [IQR], 52 months; range, 12–180 months) for the entire sample, 57 months (50 months; 12–164 months) for men, and 55 months (54 months; 12–180 months) for women. For the 304 patients that completed ≥12 months of follow-up (response rate for complete follow-up, 74.1%), the median follow-up durations were 62.5 months (47 months; 12–180 months) for all 304 patients, 63 months (47 months; 12–164 months) for men, and 61 months (48 months; 12–180 months) for women. Of the 304 patients, 252 (82.9%) were followed up in person with physical and radiological examinations at the 7 participating hospitals; 52 (17.1%) were followed up by telephone interviews, and their physical and radiological examinations were performed at local hospitals.

### Sex Differences in Demographic and Clinical Characteristics

The mean ages at diagnosis were 35.7 years (standard deviation, 13.4 years) for the entire sample of 410 patients, 37.2 years (14.1 years) for men, and 34.0 years (12.4 years) for women (sex difference, P = 0.017). There were no significant sex differences in tumor side, tumor location, Campanacci grade, pathological fracture, and surgical operation (all P > 0.05; Table 1).

### Sex Differences in Local Recurrence by Demographic and Clinical Characteristics

The local recurrence rates were 23.4% overall, 25.8% in men, and 20.7% in women (sex difference, P = 0.294; Table 2). The median times to postoperative local recurrence were 50.5 months overall, 52 months in men, and 48 months in women (sex difference, P = 0.950). No sex differences in local recurrence rate were found by age group, tumor side, Campanacci grade, pathological fracture, or surgical operation (all P > 0.05). However, there was a higher local recurrence rate in men than in women (29.1% vs 14.3%, P = 0.025) among patients with a tumor located at the distal femur. Significantly more men aged 20–39 years experienced local recurrence (33.0%) than men in other age groups (P = 0.039). Based on surgical operation, the lowest local recurrence rates were present with en-bloc marginal resection for both men (6.9%) and women (3.1%; all P < 0.0001).

### Sex Differences in the Risk Factors for Local Recurrence

The univariate analysis showed that tumor location and surgical operation were associated with local recurrence for both men and women. However, the multivariate Cox regression model showed significant sex differences in the factors associated with local recurrence. In men, tumor location and surgical operation were significant risk factors for local recurrence: using distal femur as the reference, hazard ratio (HR) 49.84 (95% confidence interval [CI], 4.42–56.26; P = 0.002) for the proximal fibula; using en-bloc marginal resection as the reference, HR 15.73 (95% CI, 4.11–60.27; P < 0.0001) for intralesional curettage and HR 9.46 (95% CI, 2.69–32.27; P < 0.0001) for curettage combined with resection. In women, surgical operation was significant for local recurrence: HR 10.76 (95% CI, 2.98–38.92; P < 0.0001) for intralesional curettage and HR 3.60 (95% CI, 1.00–13.15; P = 0.050) for curettage combined with resection (Table 3).

### Functional Outcome

**Figure 1:**
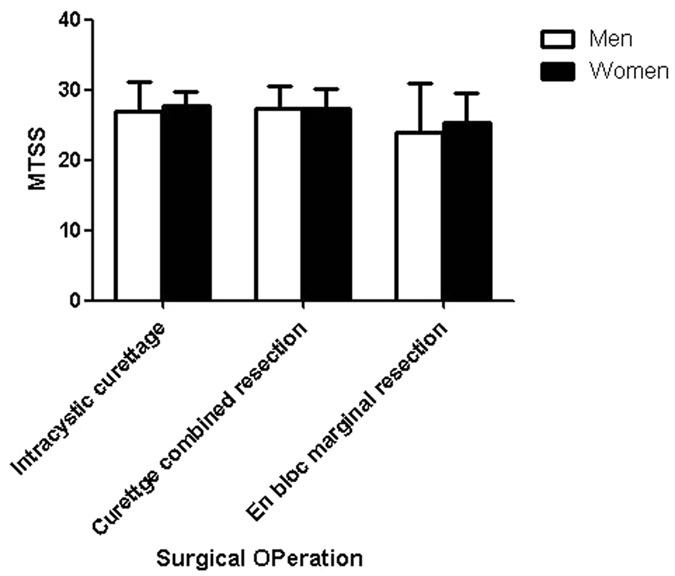
Sex difference in the median MTSS by surgical operation.

There were no significant sex differences in the Musculoskeletal Tumor Society Scores (MTSS) by surgical operation (P > 0.05; Fig. 1). The median MTSS in men were 28.0 (IQR, 17) in those treated with intralesional curettage, 28 (13) in those treated with curettage combined with resection, and 25.5 (30) in those treated with en-bloc marginal resection; the corresponding MTSS were 28.0 (6), 28.0 (10), and 27.0 (17) in women.

## Discussion

To the best of our knowledge, this is the first hospital-based, multicenter study with a large sample among individuals diagnosed with GCTB surrounding the knee; in this study, we assessed sex differences in clinical characteristics, local recurrence rate, and risk factors associated with local recurrence. Women were diagnosed at a younger age, and there higher risks of local recurrence in the proximal fibula among male patients with GCTB; moreover, higher risks of local recurrence were observed in patients who underwent intralesional curettage or curettage combined with resection in both men and women. Regarding the functional outcome, there were no significant sex differences based on surgical treatment, despite the sex differences in the risk factors for local recurrence.

In contrast to previous studies reporting a higher likelihood of GCTB in women than in men (51.5% vs 48.5%^20^ and 56% vs 44%^21^), GCTB predominately occurred in men in other studies (male:female sex ratio, 1.27–1.77:1)^7^,^10^,^22^,^23^,^24^. Similarly, in the present study, the male:female sex ratio was 1.12:1, indicating that primary GCTB around the knee predominantly occurred in men. The reason for the predominance of cases among men in Asian populations is unclear and requires further investigation. Regarding age at diagnosis, the 3-year earlier age at diagnosis among female incident cases than in male incident cases in this study might be explained by epiphyseal closure in women^17^,^18^,^24^.

A large range of local recurrence rates for GCTB has been reported previously (12–49%)^25^,^26^,^27^,^28^,^29^; however, sex differences in the local recurrence rate based on clinical features have not been reported. In the present study, overall, there was no statistically significant difference in the local recurrence rates between the male and female GCTB cases in our study (25.8% in men vs. 20.7% in women).

An association between the surgical operation and local recurrence of GCTB has been suggested previously^5^,^27^,^30^,^31^. For example, curettage was associated with an increased risk of local recurrence. Differences in local recurrence by sex based on clinical features and surgical operation have remained unclear. In the present study, there were no differences in local recurrence rate between the sexes, based on age group, tumor side, Campanacci grade, pathological fracture, and surgical operation. For age, local recurrence occurred more often for male patients aged 20–39 years than men in any other age group. However, men had a higher local recurrence rate with tumors located in the distal femur than women. The lowest local recurrence rate was observed in those treated with en-bloc marginal resection in both men and women. Moreover, the factors affecting local recurrence differed between men and women. After adjusting for age and clinical features, surgical operation and tumor location were the independent risk factors for local recurrence in men, while only surgical operation was an independent risk factor for local recurrence in women. Additional studies are needed to explain this difference.

MTSS did not differ by sex, based on surgical operation, which might be related with the few complications that occurred during the relatively short follow-up periods.

There are limitations to the present study. First, because all of the patients were recruited from 7 musculoskeletal oncology centers in local hospitals from different regions in China, there was limited representation of the general Chinese population. However, these centers covered three geographical regions (East, West, and North) among the eastern, southern, western, and northern regions of China; therefore, the data presented in this manuscript for GCTB partially represent the general situation in China. Second, because all of the patients with GCTB in this study were not recruited from the medical insurance database, we were unable to calculate the incidence and prevalence of GCTB in China. Third, given the retrospective nature and recruitment from multiple centers, there might have been differences among the institutions in identification standards for radiological data and clinical staging. However, this limitation was addressed by conducting extensive investigator training at the 7 participating centers. Fourth, data were collected for patients between 2000 and 2014; however, new techniques were not developed during this 15-year period, and the standard of diagnosis did not change. Finally, the MTSS data cannot completely reflect the postoperative function, given the relatively short follow-up periods (<12 months).

## Conclusions

To the best of our knowledge, this is the first report to describe the sex differences in clinical and demographical characteristics of primary GCTB around the knee based on a large, multicenter retrospective study in China. GCTB occurred more often in men, while women were diagnosed at a younger age than men. There was significant higher recurrence rate among men with tumor located in the distal femur than that in women. In addition, the local recurrence rate was associated with tumor location and surgical operation in men but only surgical operation in women. Finally, functional outcome did not differ by sex. These results suggest that more aggressive operations should be considered in men with GCTB in the proximal fibula.

## Materials and Methods

### Patient Selection

Between March 2000 and February 2015, patients with primary GCTB around the knee, including the distal femur, proximal tibia, proximal fibular, and patella, were recruited from the giant cell tumor of China (GTOC). The GTOC includes 7 musculoskeletal oncology centers from the northern, eastern, and northwestern regions of China: Tianjin, Shandong province, Hebei province, Inner Monggol Autonomous Region, Jiangsu province, Zhejiang province, and Shanxi province. All patients had a confirmed histological diagnosis of benign GCTB and underwent surgical treatment, in rural or urban areas. We excluded patients with a diagnosis of non-GCTB that was confirmed postoperatively, patients with recurrent GCTB, and patients with malignant GCTB owing to the differences in therapeutic methods and biological tumor behaviors between benign and malignant GCTB.

Clinical and imaging data of the primary GCTB around the knee were reviewed retrospectively to record the following information: tumor side (left or right), tumor location (distal femur, proximal tibia, proximal fibula, or patella), Campanacci staging (grade I, II, or III), pathological fracture (yes or no), and surgical treatment (intralesional curettage, curettage combined with resection, or en bloc marginal resection).

The ethics committee of Tianjin Hospital approved the study, and written informed consent was obtained from all patients during recruitment. The methods were conducted in accordance with the approved guidelines.

### Surgical Treatment

The surgical technique was based on the severity of the tumor and included intralesional curettage, curettage combined with resection, and en bloc marginal resection^23^.

Intralesional curettage was indicated for patients with a localized lesion. With this procedure, a window in the cortical bone is made, followed by resection of the mass using a series of curettes of various sizes; the residual tumor cavity is then polished with a high-speed burr until the normal cortical bone is reached and filled with an allogeneic particle bone graft to fill the window.

Curettage combined with resection was performed in patients with an extensive lesion. With this procedure, the cortical bone and soft tissue mass impossible reserved are removed, and the tumor cavity is scraped using curettes and a high-speed burr; cavitary bone defects are then filled with an allogeneic particle bone graft, and an anatomical bone plate is used for internal fixation.

En bloc marginal resection was indicated for patients with severely involved lesions. With this procedure, an osteotomy plane is confirmed based on preoperative magnetic resonance imaging, and the tumor is resected en bloc; an articulated prosthesis is used to reconstruct the knee.

### Follow-up and Functional Outcome

The patients were followed-up every 3 months for the first 2 post-operative years, every 6 months until 5 years post-operative, and every 12 months until 10 years post-operative. Telephone interviews were allowed only after 5 years of follow-up. The MTSS (total score, 30 points) was used to assess the functional outcome during the follow-up^32^.

### Statistical Methods

In all patients, the sex differences in clinical features were assessed, including the tumor side and location, Campanacci grade, pathological fracture, and surgical operation. Local recurrence rates and risk factors were analyzed in patients with at least 12 months of follow-up. Continuous variables are summarized as means (standard deviations) or medians (IQRs) and compared between sexes using Student’s *t*-tests or Mann-Whitney U tests. Categorical variables are presented as numbers (percentages), and the chi-square test was used to assess the differences in clinical characteristics, surgical operation, and local recurrence between the sexes. Kaplan-Meier estimates of survival were used for univariate analyses, while the Cox proportional hazards regression model was used for multivariate analyses of survival to determine risk factors for local recurrence in both men and women. Risk factors for local recurrence are summarized as HRs with 95% CIs. All statistical analyses were performed using SPSS version 15.0 (SPSS Inc., Chicago, IL), and two-tailed P values < 0.05 were considered statistically significant.

## Additional Information

**How to cite this article**: Hu, Y. *et al*. Sex Differences in the Recurrence Rate and Risk Factors for Primary Giant Cell Tumors Around the Knee in China. *Sci. Rep.* **6**, 28173; doi: 10.1038/srep28173 (2016).

## Figures and Tables

**Table 1:** Sex differences in demographic and clinical characteristics among patients with primary giant cell tumor of bone around the knee.

Categories	Total	Men	Women	P
Cases, n (%)	410	217 (52.9)	193 (47.1)	—
Age, years, mean (SD)	35.68 (13.38)	37.17 (14.05)	34.00 (12.41)	0.017
Age group, n (%)				0.160
<20 years	39 (9.5)	20 (9.2)	19 (9.8)	
20–39 years	235 (57.3)	117 (53.9)	118 (61.1)	
≥40 years	136 (33.2)	80 (36.9)	56 (29.0)	
Side, n (%)				0.438
Left Knee	191 (46.6)	105 (48.4)	86 (44.6)	
Right Knee	219 (53.4)	112 (51.6)	107 (55.4)	
Location, n (%)				0.355
Distal femur	213 (52.0)	111 (51.2)	102 (52.8)	
Proximal tibia	183 (44.6)	96 (44.2)	87 (45.1)	
Proximal fibula	11 (2.7)	7 (3.2)	4 (2.1)	
Patella	3 (0.7)	3 (1.4)	0	
Campanacci grade, n (%)				0.596
I	52 (12.7)	24 (11.1)	28 (14.5)	
II	160 (39.0)	88 (40.6)	72 (37.3)	
III	198 (48.3)	105 (48.4)	93 (48.2)	
Pathologic fracture, n (%)				0.340
No	271 (66.1)	148 (68.2)	123 (63.7)	
Yes	139 (33.9)	69 (31.8)	70 (36.3)	
Surgical approach, n (%)				0.568
Intralesional curettage	98 (23.9)	49 (22.6)	49 (25.4)	
Curettage combined resection	189 (46.1)	110 (50.7)	79 (40.9)	
En bloc marginal resection	123 (30.0)	58 (26.7)	65 (33.7)	

**Table 2:** Sex differences in local recurrence rates among patients with primary giant cell tumor of bone around the knee, based on demographic and clinical characteristics.

Categories	Total	Men	Women	P
Total, n (%)	71 (23.4)	41 (25.8)	30 (20.7)	0.294
Time of recurrence,	50.5 (54)	52 (56)	48 (51)	0.950
Age of Recurrence, years, mean (SD)	35.38 (12.01)	36.77 (12.43)	33.50 (11.33)	0.260
Age group, n (%)				
<20 years	4 (14.3)	1 (7.1)	3 (21.4)	0.596
20–39 years	51 (28.5)	30 (33.0)	21 (23.9)	0.177
≥40 years	16 (16.5)	10 (18.5)	6 (14.0)	0.547
Side, n (%)				
Left Knee	35 (25.7)	22 (29.7)	13 (21.0)	0.244
Right Knee	36 (21.4)	19 (22.4)	17 (20.5)	0.768
Location, n (%)				
Distal femur	34 (21.8)	23 (29.1)	11 (14.3)	0.025
Proximal tibia	35 (26.1)	17 (24.3)	18 (28.1)	0.613
Proximal fibula	2 (18.2)	1 (14.3)	1 (25.0)	1.000
Patella	0	0	0	—
Campanacci grade, n (%)				
I	8 (33.3)	4 (30.8)	4 (36.4)	1.000
II	25 (23.1)	14 (24.6)	11 (21.6)	0.713
III	38 (22.1)	23 (25.8)	15 (18.1)	0.220
Pathologic fracture, n (%)				
No	44 (26.0)	28 (30.4)	16 (20.8)	0.154
Yes	27 (20.0)	13 (19.4)	14 (20.6)	0.863
Surgical technique, n (%)				
Intralesional curettage	31 (53.4)	15 (50.0)	16 (57.1)	0.586
Curettage combined resection	34 (27.6)	22 (31.0)	12 (23.1)	0.333
En bloc marginal resection	6 (4.9)	4 (6.9)	2 (3.1)	0.419

**Table 3:** Adjusted hazards ratios with 95% confidence intervals for the risk factors of local recurrence in patients with primary giant cell tumor of bone around the knee, by sex.

Risk factors	Reference	Men	P1	Women	P2
Age	—	1.00 (0.96, 1.03)	0.970	1.00 (0.96, 1.03)	0.796
Side	Left side	0.69 (0.36, 1.33)	0.271	1.13 (0.53, 2.43)	0.753
Location	Distal femur				
Proximal tibia		0.62 (0.33, 1.39)	0.150	1.38 (0.62, 3.08)	0.432
Proximal fibula		49.84 (4.42, 562.56)	0.002	0	0.993
Campanacci grade	I				
II		0.76 (0.24, 2.36)	0.629	0.38 (0.11, 1.26)	0.379
III		1.56 (0.50, 4.90)	0.488	0.53 (0.16, 1.68)	0.276
Pathologic fracture	No	0.67 (0.32, 1.39)	0.282	0.44 (0.63, 3.27)	0.388
Surgical technique	En-bloc resection				
Intralesional curettage		15.73 (4.11, 60.27)	<0.0001	10.76 (2.98, 38.92)	<0.0001
Curettage combined resection		9.46 (2.69, 32.27)	<0.0001	3.60 (1.00, 13.15)	0.05
